# Determination of Natamycin in Turkish Yoghurt

**DOI:** 10.1155/2016/8480963

**Published:** 2016-05-03

**Authors:** Dilek Bilgic Alkaya, Ozlem Karalomlu

**Affiliations:** Faculty of Pharmacy, Department of Analytical Chemistry, Marmara University, P.O. Box 34668, Haydarpasa, Istanbul, Turkey

## Abstract

This study was aimed at developing RP-HPLC method for determination of natamycin in Turkish yoghurt. Chromatographic separation was achieved on a C8 column (150 mm × 4.6 mm × 5 *µ*m) with a mobile phase of methanol : water : acetic acid (12 : 8 : 1 v/v/v), at 1 mL/min flow rate with a detection of 303 nm. Natamycin was spiked into handmade yoghurt samples and used for validation. The method has been fully validated according to ISO 9233-2, 2007 (IDF 140-2, 2007). It was successfully applied to determination of 28 different Turkish yoghurt products. Findings dealing with the presence of natamycin in cheese samples are presented.

## 1. Introduction

Yoghurt is a fermented milk product, made from standardized milk by* Streptococcus thermophilus* and* Lactobacillus bulgaricus* [1]. These bacteria produce lactic acid during fermentation of lactose. In case of milk processed to yoghurt, the growth of lactic acid bacteria will cause pH to fall, favouring the growth of spoilage yeast. Yeasts are very common in yoghurts and can sometimes cause spoilage [2].

The use of food preservatives has increased in the food industry. These preservatives are added to prolong the shelf life of foods. In dairy products, natamycin is used as an antimicrobial food preservative, produced during fermentation by the bacterium* Streptomyces natalensis* [3]. Natamycin is a colorless, tasteless, and odorless stable compound. In addition, it is nearly insoluble in water so that it penetrates into the product, in hard cheeses <1 mm and in rindless cheese. Natamycin has also begun to be used as a preservative in yoghurt. According to the Turkish Food Codex, the maximum natamycin concentration in cheese is 1 mg/dm^2^ and should not be detectable at 2 mm depth [4]. Although yoghurt is special fermented milk product, Turkish Food Codex does not accept the use of any preservative in yoghurt [2, 5].

Therefore, in the present study, simple, economical, rapid, accurate, reproducible, precise, and fully validated HPLC method with good detection ranges for estimation of natamycin in yoghurt was developed. A number of analytical methods have been reported for the detection and quantification of natamycin including spectrophotometric [6], derivative spectrophotometric [7], and liquid chromatographic [8–14] methods. Some of these studies are with respect to compounds in some food while others are with regard to compound in drugs or biological samples. Methods of analysis in foods were based on organic solvents extraction followed by UV detection or further HPLC separation with UV detection. Although UV spectrum of natamycin shows three major absorption peaks in the range of 290–320 nm, 304 nm is the wavelength commonly used to quantify the antifungal, because of spectral interference from other compounds. Also, natamycin showed degradation in food and during the storage time; it is uneven distribution in the solution; for this reason, identification of natamycin peak was made by HPLC-DAD system in this study [15, 16].

## 2. Materials and Methods

### 2.1. Apparatus and Chromatographic Conditions

A Waters Bondapak C8 (150 mm × 4.6 mm × 5 *µ*m) was used as column in Agilent 1200 with setting of DAD detector at 303 nm. The guard column was C_18_ sep-pack. The mobile phase consisted of methanol, water, and acetic acid (12 : 8 : 1; v/v/v); flow rate was 1.0 mL/min and temperature was 25°C. Injection volume was 20 *µ*L and detector wavelength was 303 nm.

### 2.2. Chemicals and Reagents

All chemicals and reagents were of analytical grade and water was distilled and filtered through a membrane filter (0.45 *μ*m Natamycin powder was obtained from Dr. Ehrenstorfer, 16208400, 97.0%). Methanol (HPLC grade, Merck, 106018, ≥99.8%) and acetonitrile (HPLC grade, Sigma, 27225, 99.8–100.5%) acetic acid were used to prepare the dilute solution and mobile phase. Yoghurt samples from Turkey producers were obtained from trade network. All stock and working solutions were protected from light and stored in fridge at about 4°C.

### 2.3. Preparation of Standard Solution

Stock solution of natamycin (0.5 mg/mL) was prepared by dissolution in methanol. 50 mg of pure natamycin (C_33_H_47_NO_13_) was dissolved in methanol in a 100 mL one-mark volumetric flask, marked with water, mixed, and protected from light.

Stock solution of natamycin (5 mg/L) was prepared in mobile phase The concentration ranges of natamycin were 0.1 *μ*g/mL, 0.2 *μ*g/mL, 0.4 *μ*g/mL, 0.6 *μ*g/mL, and 0.8 *μ*g/mL, respectively. The calibration curve for HPLC analysis was constructed by plotting the ratio of the peak area of the natamycin.

### 2.4. Preparation of Test Solution for HPLC Analysis

Homemade yoghurt (without natamycin) varieties were used for validation and application studies. Test samples were prepared according to ISO 9223-2, 2007 [17].

### 2.5. Sample Preparation

An amount of 5 g homogenised yoghurt was weighed in conical flask and 50 mL of methanol was added. Sample was stirred for 90 min in a magnetic stirrer. 25 mL of deionized water was added and placed in the conical flask in the freezer for about 60 min. Cold extract was filtered through a folder filter paper (Macherey-Nagel 100 751/60/030); first filtrate was discarded. Filtrate was warmed on room temperature. A potion of the filtrate was filtered through a membrane microfilter of pore size of 0.45 *μ*m (Minisart RC25 17765) and then 0.20 *µ*m (Minisart RC25).

The minimum amount of test solution (filtrate) required is 20 *μ*L per injection for direct chromatographic measurement. Plot peak area or peak height was obtained for each solution on the ordinate against the natamycin concentration, inmicrograms per millilitre, on the abscissa.

### 2.6. Method Validation

*Precision*. Method precision (repeatability) was evaluated by assaying ten sets of test samples, all on the same day (intraday precision) and method precision was also determined by another person under the same experimental conditions.

*Linearity*. Six solutions were prepared containing 0.01 mg/L, 0.1 mg/L, 0.2 mg/L, 0.4 mg/L, 0.6 mg/L, and 0.8 mg/L natamycin concentration, respectively. Each solution was injected in duplicate. Linearity was evaluated by linear regression analysis.

*The Limit of Detection (LOD) and Limit of Quantitation (LOQ)*. It is the lowest concentration of analyte in the sample that can be determined with the acceptable precision and accuracy under stated experimental condition. The solution was injected three times and the signal and the noise for each injection were recorded. Each signal-to-noise ratio was then calculated and averaged. The concentration of the solution was used to determine the detection limit if the average S/N ratio is between 3 and 10. Also, LOQ value was calculated from the calibration curve using equation LOQ = 10 × SD/*b* (where SD is standard deviation of intercepts of calibration and *b* is slope of corresponding calibration curve). The LOD and LOQ values were found to be 0.499 mg/kg and 0.403 mg/kg for yoghurt samples, respectively.

*Accuracy*. Accuracy was assessed by determination of the recovery of the method at two different concentrations (3 mg/kg and 6 mg/kg of the test solution concentration).

*Specificity and System Suitability*. The stability of the chromatographic system was tested before each stage of validation. 20 replicate injections of sample preparation were injected and number of theoretical plats, tailing factor, and relative standard deviation of peak area were determined.

### 2.7. Statistical Analysis

Statistics for data analysis were done with Excel programme. Student's* t*-test and ANOVA* F* test were used to determine significant differences between dairy brands.

## 3. Results and Discussion

Natamycin is an antimicrobial food additive against yeast and moulds. For this reason, the use of natamycin has increased in food industry. Yoghurt is fermented milk product and has a short shelf life. Although use of natamycin is restricted in yoghurt, some producers could add natamycin as preservative [5, 18]. However, it is accepted to use safe food additive in some countries [2]. In this paper, an analytical HPLC method for assay of natamycin in yoghurt products was developed and validated. The proposed method gives good resolution within a short analysis time. The simultaneous determination of the chees samples was performed on a C8 column of (150 mm × 4.6 mm) dimension and 5 *μ*m of particle size. A mixture of methanol, water, and acetic acid (12 : 8 : 1; v/v/v) was used as mobile phase with flow rate of 1 mL/min. The effluent was monitored at 303 nm. Natamycin contains strong chromophores and shows most intense absorption at 303 nm [6]. According to ISO 9223-2, 2007 [17], preparation of test sample system suitability test can be defined as a test to ensure that the method can generate results of acceptable accuracy and precision. The requirements for the system suitability are usually designed after method development and validation have been completed. System suitability test results were reported in Table 1.

The components of the cheese samples did not show any interference at 304 nm and no detector signal was produced during the analysis.

**Figure 1:**
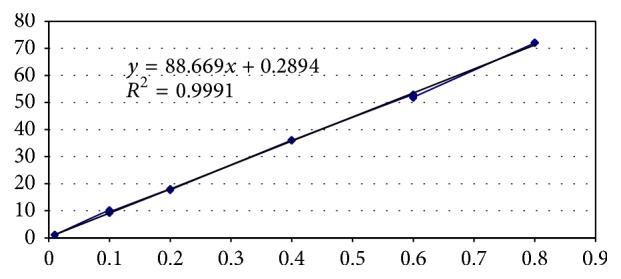
Linearity of natamycin.

The calibration curves (DAD) were constructed with five concentrations including the lower limit of quantification (LOQ) ranging from 0.01 to 0.8.  Table 2 includes the calibration data and related validation parameters. The regression analysis was performed, showing the equation: *y* = 88.669*x* + 0.2894. Correlation coefficient was 0.9991 (Figure 1). This shows good linearity of the method. Retention time was 2.98 min; the limit of detection (LOD) and limit of quantification (LOQ) were determined as 0.320 mg/kg and 0.403 mg/kg, respectively, by taking 3 and 10 times the standard deviation using the slope of calibration curve of natamycin, respectively. Recovery experiments were performed at three concentrations of 104% and the values of relative standard deviation (RSD) were 0.562%. Both the standard natamycin mixture and the sample showed good linearity in the tested range. By applying linear regression analysis, the slope, intercept, and regression standard deviation were calculated.

**Figure 2:**
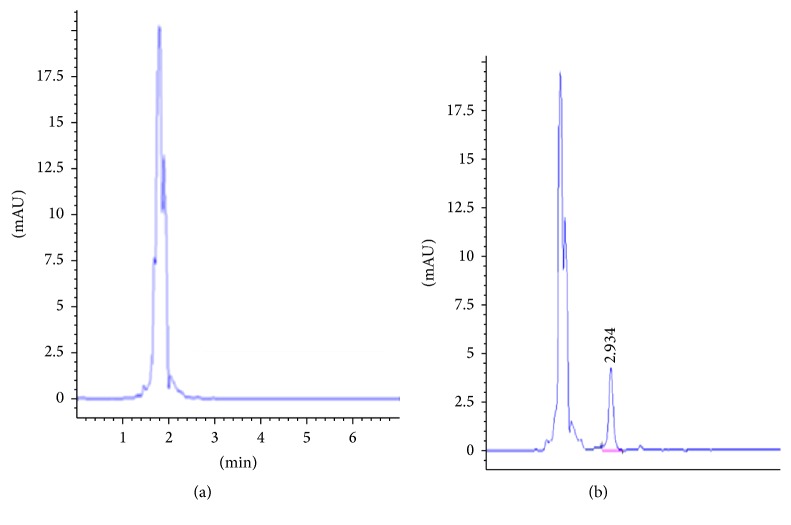
Chromatogram obtained from the mobile phase (a) and yoghurt (b).

Figure 2(b) shows a typical chromatogram obtained for analysis of spiked natamycin in yoghurt. As shown in Figure 2(b), the substances formed well shaped natamycin peak that was well separated from the mobile phase.

**Figure 3:**
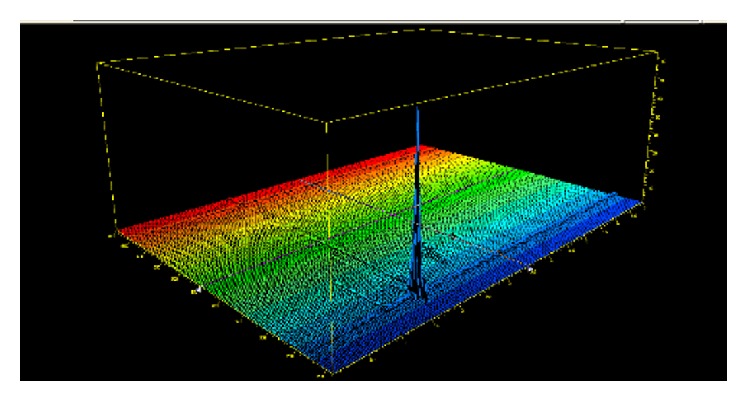
3D DAD spectrum of natamycin.

**Figure 4:**
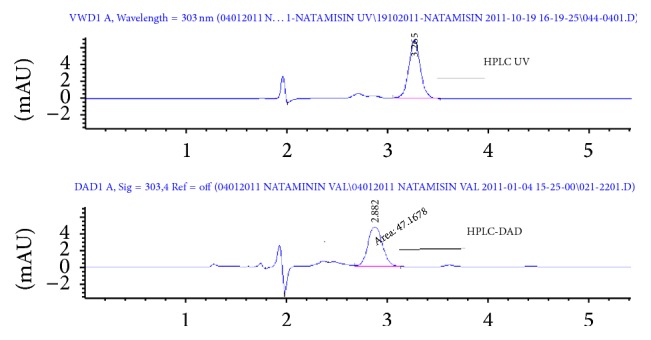
Natamycin peaks for UV and DAD detectors.

The accuracy of the developed method was carried out by adding the known amount of natamycin pure drug to the preanalyzed yoghurt sample and subjected to the proposed method. Results of recovery study are shown in Table 3. The study was done at 3 mg/kg and 6 mg/kg of test concentration levels. All the results indicate that the method is highly accurate. A specimen HPLC DAD chromatogram and 3D spectrum of a sample solution are shown in Figures 3 and 4.

Precision of the assay was determined by intraday and intermediate assay of the developed method. Intraday analysis refers to the use of the analytical procedure in a laboratory over a short period of time which was evaluated by assaying 10 sample solutions for each analyst, at the final concentration corresponding to 0.6 mg/kg of natamycin during the same day. Intermediate assay was done by two different analysts. The interday precision (repeatability) of the method was found as 1.874% and 3.442% RSD values for UV and DAD detections. Recovery studies were also carried out to determine accuracy and precision of the proposed method. The recovery procedure was carried out by spiking already analyzed samples of homogenized yoghurt with known concentrations of standard solution of natamycin. The results of the recovery analysis are shown in Table 3. According to Student's *t*-test, the computed value is less than the table value and and accuracy parameter has been validated.

Analysis of food preparations was carried out according to steps mentioned in Sample Preparation. Results were fitting with the label claims for each of the substances. The results obtained by two different analysts were compared with ANOVA *F* test and they were not statistically different.

In Turkey, few companies use modern techniques in the production of yoghurt; generally, yeast and moulds can occur in yoghurt after 1 week of storage; also, use of basic technology could cause problem in the shelf life of yoghurts. According to Turkish Food Codex, fermented dairy products should not contain any preservatives; some producers use natamycin as a preservative to prevent the occurrence of yeast and moulds in yoghurt [5]. A total of twenty-eight yoghurt samples purchased from the supermarkets were analyzed to evaluate the natamycin contents and results are listed in Table 4. Natamycin was detected in all yoghurt samples and it is not acceptable according to Turkish codex.

## 4. Conclusion

The RP-HPLC method enables the determination of natamycin with good accuracy and precision, either in laboratory-made yoghurt samples or in the commercial yoghurt samples. The developed method gives a good resolution in 7 min. High recovery shows that the method is free from the interference of the commonly used additives in the yoghurt product.

## Figures and Tables

**Table 1:** System suitability parameters of the proposed RP-LC method.

Parameters	DAD
Retention time (min)	2.98 ± 0.2
Theoretical plates (N)	4385 ± 383
Tailing factor	0.82 ± 0.01
Peak wavelength	0.18 ± 0.01

**Table 2:** Statistical evaluation of the calibration data of natamycin.

Linearity	Natamycin (DAD)	
	Concentration	Peak area
Standard solution	0.01	1.072
	0.1	9.668
	0.2	17.814
	0.4	36.028
	0.6	52.197
	0.8	72.049

Slope	88.669	

SE of slope	1.219	

Intercept	0.2894	

SE of intercept	0.0544	

Each value is the mean of 3 experiments		

**Table 3:** Accuracy and precision study of natamycin samples.

Detector	Parameters					
	Mean assay (mg/kg)	Standard Deviation	RSD (%)	LOD (mg/kg)	LOQ (mg/kg)	Recovery (%)
DAD	0.54	0.051	3.256	0.320	0.403	89

**Table 4:** Natamycin content in yoghurt samples.

Sample number	Natamycin concentration (mg/kg)
1	1.23
2	<LOQ
3	<LOQ
4	<LOQ
5	<LOQ
6	<LOQ
7	1.34
8	<LOQ
9	<LOQ
10	<LOQ
11	1.71
12	1.71
13	1.71
14	1.71
15	<LOQ
16	<LOQ
17	<LOQ
18	<LOQ
19	<LOQ
20	<LOQ
21	<LOQ
22	<LOQ
23	<LOQ
24	<LOQ
25	1.15
26	<LOQ
27	4.89
28	<LOQ
